# A human reproductive approach to the study of infertility in chimpanzees: An experience at Leon’s Zoological Park, Mexico

**Published:** 2016-09-15

**Authors:** Raul Eduardo Piña-Aguilar, Janet López-Saucedo, Lilia Ivone Ruiz-Galaz, José de Jesús Barroso-Padilla, Mayra Celina Gallegos-Rivas, Claudia González-Ortega, Antonio Martin Gutiérrez-Gutiérrez

**Affiliations:** 1 *Faculty of Medicine, Autonomous University of Yucatan, Merida, Mexico;*; 2 *Institute of Sciences in Human Reproduction Vida, Leon, Mexico;*; 3 *Leon’s Zoological Park, Leon, Mexico.*; † *Present address: School of Medicine, Medical Sciences and Nutrition, University of Aberdeen, Aberdeen, Scotland.*

**Keywords:** Chimpanzee, Cryptozoospermia, Cytogenetic analysis, Electroejaculation, Ultrasound

## Abstract

Great apes are mammals close to humans in their genetic, behavioral, social and evolutionary characteristics and new genomic information is revolutionizing our understanding of evolution in primates. However, all these species are endangered. While there are many global programs to protect these species, the International Union for Conservation of Nature (IUCN) projects that in a near future the wild populations will decrease significantly. Nowadays, the relevance of captive populations of great apes is becoming critical for research and understanding of pathophysiology of diseases. In this report, the evaluation of infertility in a group of captive chimpanzees maintained at Leon’s Zoological Park using a human infertility protocol is described. Our results suggested that infertility in this group was due to low hormonal levels and sperm alterations in the male characterized by hormonal assessment and a sperm sample obtained by electroejaculation and cryopreserved using human protocols. In the females, it was demonstrated that it is possible to follow the follicular cycle using non-invasive methods based on morphological changes in genitalia, detection of blood in urine and measurement of hormones in saliva samples; concluding that fertility in females was normal. Also, we demonstrate that human artificial insemination procedures may be applied. Our human approach was successful in finding the infertility cause in this group of captive chimpanzees. In countries with limited resources, collaboration of zoos with human infertility clinics can be beneficial for research and management of reproductive aspects of great apes.

## Introduction

Chimpanzees are charismatic species, well known to the public because of their physical and behavioral features are similar to humans. They are exhibited in many zoos around the world and controversially some individuals took part in entertainment shows in television programs, movies, circuses and even some are maintained as pets. However, in general, public is not aware of the conservation issues around them, such as the existence of four subspecies of chimpanzees: West African chimpanzee (Pan troglodytes verus), Nigeria-Cameroon chimpanzee (P. t. ellioti), Central chimpanzee (P. t. troglodytes) and Eastern chimpanzee (P. t. schweinfurthii).^1^ The natural habitat of chimpanzees is discontinued across 22 countries in Central Africa. Although many sites where chimpanzees live are protected World Heritage Sites, the future of this species is imperil principally by human activities, such as poaching and habitat destruction.^2^

Contrasting to the apparent “easy” reproduction of chimpanzees in the highly controlled conditions of research centers, little is known about the reproduction or the fertility features of these animals in the wild. Almost all the knowledge about reproduction of this species came from studies performed in research colonies mainly at Yerkes National Primate Research Center in  United States,^3^^-^^5^ but United States National Institutes of Health (NIH) imposed a breeding moratorium in 1990s.^6^ The National Institutes of Health and Gabon used to maintain “legal” research in chimpanzees,^6^ however over the past few years all invasive chimpanzee research in United States was baned.^7^ Recently, the status of chimpanzees maintained in captivity in United States was changed to an endangered status similar to the wild populations.^8^ Future research related to chimpanzee reproduction will depend completely on wild populations and ex-situ populations maintained principally at zoos.

Assisted reproduction techniques are an option to maintain genetic diversity and improve population management of endangered species, especially in captivity. However, the capability of zoos, especially Latin American institutions, to perform these activities is limited due to lack of resources, infrastructure and knowledge. In the case of apes, their genetic and reproductive similarities to humans, allow the collaboration with human infertility clinics which has the tools and resources to carry out this kind of research.

The aim of the present work is to describe the collaborative experience between a Mexican zoo and a human infertility clinic in a reproductive assessment of the group of chimpanzees at the Leon’s Zoological Park in Central Mexico using a human infertility approach.

## Materials and Methods

All procedures in animals were performed according to the NOM-062-ZOO-1999 which regulates the use of laboratory animals in Mexico and the Chimpanzee Care Guidelines of the American Zoological Association. Leon’s Zoological Park at the central area of Mexico maintains four chimpanzees (P. troglodytes), that have been living together for more than 15 years without presenting reproduction. One male (M1) came from a circus, he lost an arm trying to escape from it and has a lack of interest in females, the other male (M2) is castrated. The females (F1 and F2) are relatively young (21 and 29 years old, respectively), they show reproductive behavior and were considered presumably fertile. We performed a reproductive analysis using human infertility protocols, trying to find the cause of infertility in this group.

Reproductive assessment in females. We began our program with the characterization of estrus cycles in the females. External anatomy and behavioral observations were performed during three months. Later, we conditioned the females for non-invasive saliva sampling for hormonal determination of β-estradiol and luteinizing hormone (LH) as markers of ovulation and performed determinations of blood in urine with urinary test strips to detect menstrual bleeding.

Invasive procedures. During biannual administration of tuberculin (PPD) for diagnosis of tuberculosis, we decided to include a complete reproductive analysis to three chimpanzees (F1, F2 and M1). For this purpose, we anesthetized them with a dose of 5 mg kg^-1^ of tiletamine/ zolazepam (Zoletil®, Virbac, Carros, France). Blood samples were taken from the male for determination of testosterone, follicle-stimulating hormone (FSH) and LH, blood cells count and karyotype. The next step was semen collection, first, a Foley's catheter (8F) was introduced for drainage of urine and electroejaculation was performed with an electroejaculator probe of 2.30 cm of diameter and 18 cm long with a circular electrode (Bailey electro-ejaculator; Western Instrument, Denver, USA). We used a protocol that consisted of 31 cycles (1 sec of voltage and 1 sec of rest) in two repetitions with a voltage of 6 and 12 V, respectively. The semen sample coagulated inside the Foley’s catheter was transported to the laboratory in HTF-HEPES media (LifeGlobal, Guilford, USA); it was incubated at 37 ˚C until partial liquefaction and centrifuged. The concentration was determined with a Makler's chamber (Fertility Tech, Rockaway, USA) and the morphology was analyzed with Papanicolau's stain using World Health Organization (WHO) human sperm criteria.^9^ The sample was frozen using Tris-yolk of egg medium (Irvine Scientific, Irvine, USA) in cryovials. Blood samples were taken from females for determination of LH, FSH, β-estradiol and progesterone, blood cells count and chromosomal analysis.

Reproductive hormones and cytogenetic analyses. Both serum and saliva samples were processed by a chemilumiscence immunoanalyzer (Immulite 100, Siemens, Munich, Germany) using human kits specific for each hormone. Luteinizing hormone and β-estradiol levels were also measured by radio immunoanalysis in an external laboratory. The cytogenetic analysis was performed using human standard techniques (culture of lymphocytes in RPMI 1640 media, stimulation with PHA and harvesting at 72 hr, GTG banding) and compared with the ideogram described for the chimpanzee.^10^

**Fig. 1:**
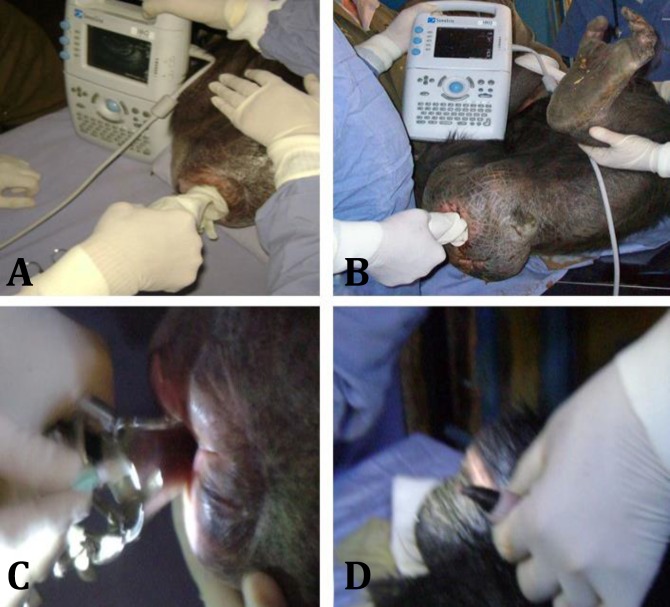
Reproductive assessment of chimpanzees. A) Transvaginal ultrasound of Female 1. B) Transvaginal ultrasound in Female 2 showing genital swelling associated to a postovulatory state. C) Sham artificial insemination in Female 1. D) Erection during electroejaculation procedure in Male 1

Ultrasound and sham artificial insemination. A transvaginal ultrasound scanning (Fujifilm SonoSite Inc., Washington, USA) was performed to evaluate the reproductive organs and obtain correlation with hormone levels (Figs. 1A and 1B). Additionally, in both females, a sham artificial insemination was performed visualizing the cervix with vaginoscopy using an intrauterine insemination catheter (CCD Laboratories, Paris, France), (Fig. 1C).

## Results

It was possible to characterize complete cycles (menstruation and genital swelling) with the observation of behavioral and genital changes in both females. In one of them (F1), it was possible to take samples of saliva almost daily during the period of swelling to determine peak of LH in this period (Fig. 1B).

In the male, the first two cycles of electroejaculation using 6V did not generate muscular contraction nor erection, the change to 12V produced an erection and adequate pelvic muscle contractions (Fig. 1D). However, the last cycle did not produce a semen sample, immediately we introduce a Foley catheter in which the seminal fluid and a small clot were visible, the catheter was transported to the laboratory. The concentration was 10^6^ sperms in the complete ejaculate obtained after centrifugation of transport medium, corresponding to a cryptozoospermia. The sperm morpho-logical evaluation found 65.00% abnormal spermatozoa (64.00% alterations in head, 32.00% in middle piece and 23.00% in tail), using WHO human criteria.^9^

**Fig. 2:**
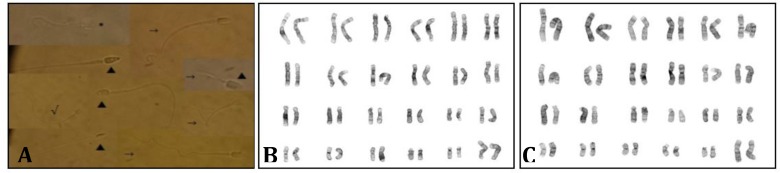
Sperm morphology analysis and cytogenetic studies. A) Composite image of normal (√) and abnormal spermatozoa, including double heads (*), abnormal tails (→) and heads (▲) (1000x). B) and C) Karyotypes of females showing normal chromosomal complement 48,XX[20].

A composite image of spermatozoa is shown in Figure 2A. The post-freezing viability of spermatozoa was 0.00%. In the case of females, anesthesia procedure was performed two days after the detection of the peak of LH in F1 and two days before menses in F2. The vaginal ultrasound revealed a uterus in ante-version with normal echogenic characteristics in each female; in F1 coinciding with a postovulatory stage, the presence of a luteal body was detected, in F2 ovaries without follicles of more of 3 mm were detected and no alteration was detected in the annexes. It was impossible to introduce the insemination catheter by vaginoscopy in F2. In the other female (F1), catheter introduction was easy, despite of genital swelling, suggesting that future insemination using human traditional catheters will be possible.

The results of hormonal determinations are shown in Table 1.There was no correlation between serum and saliva levels of β-estradiol measured by radio immunoassay and chemiluminiscence; but for LH there was a correlation between the levels in saliva and serum. Therefore, the utility of measuring β-estradiol in saliva is not useful as a predictor of ovulation using our laboratory techniques. In both females, a correlation between hormone levels, behavior/anatomic estrous cycle and ultra sonographic image was found.

**Table 1:** Hormonal values in studied animals

**Hormone**	**Technique**	**Male 1 **	**Female 1 ** (* Saliva samples **)**	**Female 2**
**Luteinizing hormone**	Chemiluminiscence	1.30 mU mL^-1^ -	22.90 mU mL^-1^ 0.45 mU mL^-1^*	1.60 mU mL^-1^
	Radioimmunoassay	-	18.15 mU mL^-1^	1.32 mU mL^-1^
**Follicle-stimulating hormone**	Chemiluminiscence	0.45 mU mL^-1^	19.80 mU mL^-1^	12.60 mU mL^-1^
**Testosterone**	Chemiluminiscence	390.00 ng dL^-1^	-	-
**β-Estradiol**	Chemiluminiscence	-	30.70 pg mL^-1^ 242.00 pg mL^-1^*	81.70 pg mL^-1^
	Radioimmunoassay	-	6.93 pg mL^-1^	35.42 pg mL^-1^
**Progesterone**	Chemiluminiscence	-	2.30 ng mL^-1^	0.20 ng mL^-1^

Furthermore, M1 presented low levels of FSH and testosterone (using human normal values), giving hormonal support to the cryptozoospermia founded and no post-freezing viability. However, it is not possible to reject that these results were produce by electroejaculation. The cytogenetic analysis concludes that both females possess a normal karyotype (48, XX) (Figs. 2B and 2C), discarding a chromosomal aberration as infertility cause in females. In male, no metaphases were retrieved after 72 hr culture, so it was not possible to determine karyotype.

## Discussion

In this study, we decided to address the absence of reproduction in a group of chimpanzees using a human infertility approach that is routinely performed in the private practice of authors. The guidelines for the study of male infertility of the American Society of Reproductive Medicine consider as essential to perform  physical examination, semen and hormonal analyses as basic tests and proposemore specialized tests such as karyotype for complementing the evaluation of male infertility.^11^ In females, the recommended first step is the evaluation of ovulation that includes hormonal evaluation, history of menstrual bleeding and serial ultrasound.^12^ Additional recommended tests are related with anatomical factors, such as tubal permeability assessement by hysterosalpingography or laparoscopy.

The assessment of reproductive status of chimpanzees using techniques developed for humans represents particular challenges. First, the ejaculate of male chimpanzee is different from humans because it contains a protein in seminal plasma that coagulates semen completely at emission and it is not completely dissolved after incubation.^13^

Furthermore, it cannot be obtained by auto-masturbation. In this case, we decided to use electro-ejaculation for obtaining the semen samples. In female chimpanzees, the ovary cycle is 36 days,^14^ longer than humans, but reproductive cycle can be assessed by genital swelling (Figures 1A and 1B) and 3 days of menstrual bleeding. We tested the use of conditioning with positive reinforcement to obtain saliva samples (Table 1), daily evaluation of genital characteristics and urine for bleeding. Functional tests of ovary such as clomiphene test or serial vaginal ultrasound,^12^ cannot be applied. As was mentioned, the development of the techniques of analysis of fertility and assisted reproduction techniques in chimpanzee were developed in Primate Research Centers. However, these techniques are not always applicable in zoos, where non-invasive procedures are preferred and the animal welfare is paramount.

Our results suggest that the cause of infertility in the group of chimpanzees is low hormonal level and sperm alterations in male (M1), in addition to their obvious low libido and disruptive behavior. Also, it was demonstrated that it is possible to monitor the follicular cycle with anatomical features and non-invasive determination of LH in females. Apparently, human cryopreservation protocols are useful for chimpanzee semen obtained by an artificial vagina,^15^ supporting that poor sperm viability found in M1 may be related with his testicular condition and not with the cryopreservation protocol.

In Mexico, chimpanzees maintained at zoos are not part of an integrated management as it is performed in United States of America and European zoos through species survival plans. Although no anti-conceptive measurements are in place, few Mexican zoos reproduce great apes successfully. Therefore, one option to continue our work is to establish collaborations with other Mexican zoos to obtain samples of semen and perform an artificial insemination in our females; but this requires an extensive discussion about implications for the hosting institutions and animal welfare.

In conclusion,this study demonstrated that a collaboration between human infertility clinics and zoos may be beneficial in the assessment of reproductive status in great apes and the possible application of assisted reproductive techniques in Latin-America and others countries with limited resources for wildlife research and conservation.
